# The Neuroprotective Potential of Vitamin D_3_

**DOI:** 10.3390/nu17203202

**Published:** 2025-10-12

**Authors:** Jacek Pietruszkiewicz, Katarzyna Mrozek, Mateusz Zwierz, Agata Wińska, Maria Suprunowicz, Aleksandra Julia Oracz, Napoleon Waszkiewicz

**Affiliations:** Department of Psychiatry, Medical University of Bialystok, pl. Wołodyjowskiego 2, 15-272 Białystok, Poland; 39913@student.umb.edu.pl (K.M.); 39995@student.umb.edu.pl (M.Z.); agataawinska@gmail.com (A.W.); maria.suprunowicz@sd.umb.edu.pl (M.S.); aleksandra.oracz@sd.umb.edu.pl (A.J.O.); napoleon.waszkiewicz@umb.edu.pl (N.W.)

**Keywords:** vitamin D, neuroprotection, Alzheimer’s disease, Parkinson’s disease, multiple sclerosis, autism spectrum disorder

## Abstract

Vitamin D_3_ plays a pivotal role not only in bone health but also in the functioning of the nervous system, particularly in the context of age-related neurodegenerative diseases such as Alzheimer’s disease, multiple sclerosis, and Parkinson’s disease. Vitamin D_3_ deficiency has been associated with cognitive decline, heightened inflammation, and shortened leukocyte telomere length, which may contribute to accelerated cellular aging. Therapeutic interventions involving vitamin D_3_ have been reported in selected clinical studies and meta-analyses to potentially enhance cognitive function, decrease amyloid β biomarkers, and prolong telomere length, although heterogeneity remains across study designs and populations. Furthermore, vitamin D_3_ has been shown to influence the expression of genes implicated in DNA repair and oxidative stress response, including *NRF2*, *OGG1*, *MYH*, and *MTH1*. This narrative review synthesizes current knowledge on the molecular mechanisms of vitamin D_3_ action in the context of neuroprotection and discusses potential directions for future research, including its possible therapeutic applications in neurodegenerative diseases.

## 1. Introduction

Vitamin D_3_ plays a pivotal role in numerous physiological processes (Table 1).

Vitamin D_3_ deficiency is increasingly recognized as a contributing factor in the pathogenesis of a growing spectrum of diseases, including endocrine and metabolic disorders, musculoskeletal conditions, connective tissue diseases, chronic kidney disease, malabsorption syndromes, obesity, cancer, and central nervous system (CNS) disorders [22]. The first step of its metabolic activation involves the skin, where 7-dehydrocholesterol is converted to cholecalciferol (vitamin D_3_) under ultraviolet radiation [23]. Vitamin D_3_ is then transported by vitamin D-binding protein (DBP) to the liver, where 25-hydroxylation, primarily catalyzed by CYP2R1, occurs [23]. The resulting 25-hydroxyvitamin D (25(OH)D) undergoes further 1α-hydroxylation in the kidneys, catalyzed by CYP27B1, to form the biologically active metabolite 1,25-dihydroxyvitamin D (1,25(OH)_2_D) [23]. This process is stimulated by parathyroid hormone (PTH) and inhibited by calcium, phosphate, and fibroblast growth factor 23 (FGF23) [24]. Additionally, extrarenal synthesis of 1,25(OH)_2_D can occur in keratinocytes and macrophages, stimulated by cytokines such as tumor necrosis factor-alpha (TNF-α) and interferon-gamma (IFN-γ) [25]. Vitamin D metabolites are also directed toward catabolic pathways. Hydroxylation at the C-24 position, mediated by CYP24A1, leads to inactivation and degradation [26]. Many human cell types express vitamin D receptors (VDRs) [27]. Upon activation by 1,25(OH)_2_D, VDRs regulate gene expression through vitamin D response elements (VDREs) in target genes [28].

Vitamin D has also been implicated in neuroprotection [29]. Its deficiency has been associated with increased risk of neurological disorders, and its role in CNS repair processes is an active area of research. Beyond renal tissue, 25(OH)D_3_ can be converted to 1,25(OH)_2_D_3_ in the brain [24]. Both forms cross the blood–brain barrier, and 1α-hydroxylation can occur locally within brain tissue [24]. Moreover, 25-hydroxylation of cholecalciferol occurs in the brain, similar to the liver [30]. Astrocytes express VDRs, which, when stimulated by 1,25(OH)_2_D, initiate gene transcription [31]. Conversely, excess vitamin D leads to VDR downregulation [30]. Vitamin D also exerts rapid, non-genomic actions via autocrine and paracrine mechanisms. These involve activation of membrane-associated receptors, including PDIA3 [32].

**Figure 1:**
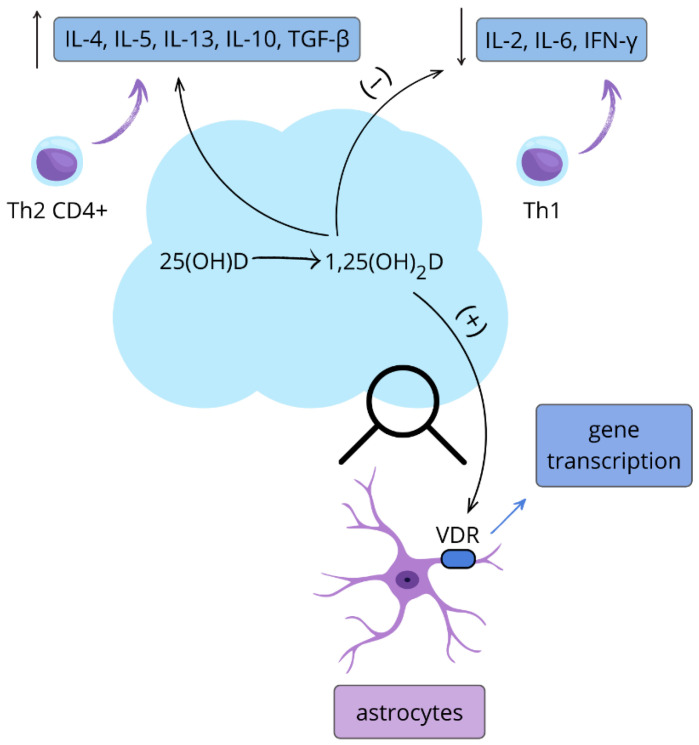
The role of active vitamin D in the nervous and immune systems. 25-hydroxyvitamin D (25(OH)D) is metabolized to its active form 1,25-dihydroxyvitamin D_3_ (1,25(OH)_2_D_3_), which shifts CD_4_-positive T helper lymphocytes toward the Th2 phenotype by increasing interleukin-4 (IL-4), interleukin-5 (IL-5), and interleukin-13 (IL-13) production, suppresses Th1 cytokines interleukin-2 (IL-2), interleukin-6 (IL-6), and interferon-gamma (IFN-γ), and activates vitamin D receptors (VDR) on astrocytes to induce gene transcription; increase (↑), decrease (↓). This figure is original.

Given the widespread presence of VDRs in CNS cells, an immunomodulatory role of vitamin D has been proposed [33]. VDRs are also present on monocytes, antigen-presenting cells (APCs), and activated lymphocytes, and vitamin D_3_ enhances the expression of both 1α-hydroxylase and 25-hydroxylase in these cells [34,35]. Furthermore, 1,25(OH)_2_D_3_ suppresses the secretion of IL-2, IL-6, and IFN-γ by Th1 lymphocytes, while promoting a shift in CD4+ T cells toward the Th2 phenotype, increasing the production of IL-4, IL-5, and IL-13 [36,37]. Vitamin D_3_-conditioned dendritic cells further inhibit IFN-γ production by Th1 cells and promote the differentiation of CD_4_+ T cells into regulatory T cells (Tregs), which secrete anti-inflammatory cytokines IL-10 and TGF-β [38] (Figure 1). It should be noted, however, that vitamin D’s immunomodulatory effects are not uniform across all physiological and pathological states. Depending on disease phase, cytokine milieu, seasonality, and concomitant treatments, vitamin D may upregulate certain cytokines while simultaneously suppressing pathogenic pathways. This context-dependent profile is important for interpreting data from disease-specific studies presented later in this review.

The supplementation of vitamin D_3_ should be tailored to individual factors such as age, body weight, diet, sun exposure, and lifestyle. International recommendations vary: in the United States, the recommended daily intake is 600 IU for individuals aged 1–60 years, while in the United Kingdom, a universal supplementation of 400 IU/day is suggested for individuals ≥ 4 years [39]. According to the Endocrine Society, adults may safely receive up to 4000 IU/day to prevent deficiency, whereas the European Food Safety Authority sets the tolerable upper intake level at 4000 IU/day for adults and adolescents and proportionally lower values for children [40]. National guidelines may differ. For example, Polish recommendations (updated in 2023) advise daily supplementation of 1000–2000 IU (25–50 µg) for adults aged 19–65 years with normal body weight, with upper limits of 4000 IU/day for adults, adolescents, and pregnant or breastfeeding women [41]. Children aged 1–10 years are advised to receive up to 2000 IU/day, while infants below 1 year should not exceed 1000 IU/day. In cases where adequate serum 25(OH)D concentrations (≥30 ng/mL; 75 nmol/L) cannot be achieved through sun exposure or standard supplementation, calcifediol (25-hydroxyvitamin D_3_) at 10 µg/day may be considered [41]. Vitamin D toxicity is uncommon but possible. Serum levels above 100 ng/mL (250 nmol/L) are associated with an increased risk of adverse effects according to Polish guidelines, whereas U.S. sources consider > 150 ng/mL (375 nmol/L) potentially toxic [39,41]. Manifestations include hypercalcemia, hyperphosphatemia, and hypercalciuria, with chronic toxicity leading to extraosseous calcifications (kidneys, blood vessels, myocardium, lungs, skin) and electrocardiographic changes such as QT shortening [42]. For these reasons, supplementation should follow national and international guidelines, accounting for regional differences in sun exposure and dietary intake, while avoiding excessive dosing.

This narrative review is subject to important limitations that must be considered when interpreting the presented findings. First, as a narrative review, it does not apply systematic inclusion or exclusion criteria, nor does it critically appraise methodological quality. The included studies varied in sample size, methodological rigor, and supplementation regimens, which reduces the consistency of the conclusions. Reported discrepancies may stem from differences in the sensitivity and specificity of measurement methods, heterogeneity of patient populations (e.g., different deficiency thresholds, dosing schedules, outcome measures), and lack of adjustment for confounding factors such as age, sex, BMI, metabolic status, sun exposure, smoking, or concomitant medications. Furthermore, findings derive from both observational and interventional studies, limiting causal inference. Despite these limitations, the reviewed literature consistently links vitamin D_3_ with neuroprotective mechanisms and suggests beneficial clinical effects in selected populations. Nevertheless, further well-designed studies are warranted, applying standardized definitions of deficiency, uniform 25(OH)D assays, and clinically meaningful endpoints, while controlling for key confounders.

## 2. Materials and Methods

The literature search was conducted using PubMed, Web of Science, and Google Scholar. The search covered articles published between 1969 (the earliest identified study on vitamin D and neuropsychiatric mechanisms) and 2025, with the last search performed on 22 September 2025. The following keywords and combinations were used: “vitamin D_3_,” “cholecalciferol,” “neuroprotection,” “Alzheimer’s disease,” “Parkinson’s disease,” “multiple sclerosis,” “autism spectrum disorder,” “neuroplasticity,” “neuroinflammation,” “oxidative stress,” “telomere,” “biomarkers,” and “supplementation.” Both original research and review articles were considered during the initial screening. References were managed using Zotero (version 7.0.14, 64-bit; Corporation for Digital Scholarship, Fairfax, VA, USA), which was also used to remove duplicates generated due to overlapping search terms. Since this work is a narrative review, no formal inclusion or exclusion criteria were strictly applied. During the screening process, non-English publications, conference abstracts, and papers with insufficient methodological details were excluded. The final selection included peer-reviewed studies that directly addressed the neuroprotective role of vitamin D_3_ or provided mechanistic, clinical, or translational insights relevant to the topic.

## 3. Neuroplasticity and Vitamin D

**Figure 2:**
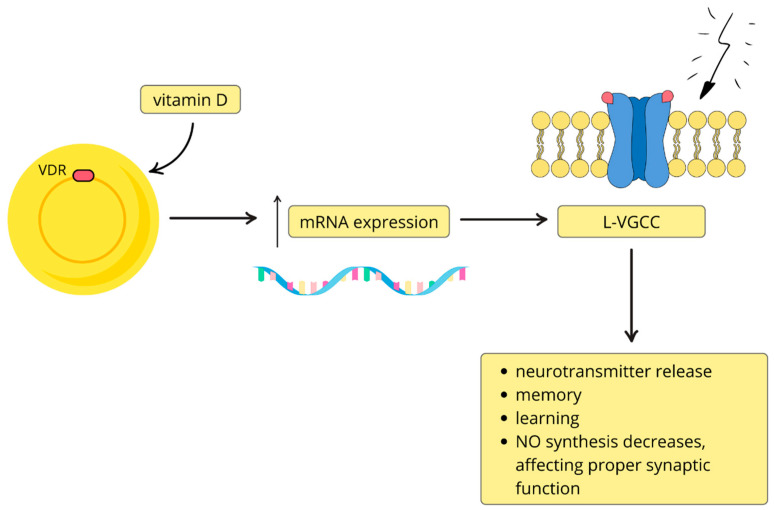
Vitamin D and calcium channel regulation in the nervous system. By activating nuclear vitamin D receptors (VDR), vitamin D increases (↑) the expression of messenger RNA (mRNA) encoding subunits of L-type voltage-gated calcium channels (L-VGCC), leading to neurotransmitter release, participation in learning and memory processes, and proper synaptic function. Modulation of L-VGCC activity is particularly important for synaptic plasticity, which underlies learning and memory. This figure is original.

Neuroplasticity, defined as the formation of new synapses, the elimination of existing ones, or the modification of their properties, is influenced by vitamin D through several molecular mechanisms [43,44]. Experimental models show that vitamin D regulates the expression of genes involved in synaptic remodeling, including neuromodulin and drebrin, whose abnormal expression has been reported in schizophrenia [45,46]. Vitamin D further promotes neuroplasticity by influencing dopaminergic, serotonergic, and glutamatergic signaling, as well as calcium-dependent neuronal processes [47]. Through activation of nuclear vitamin D receptors, it increases transcription of mRNA encoding subunits of L-type voltage-gated calcium channels (L-VGCCs), and via non-genomic mechanisms enhances calcium influx through kinase-dependent phosphorylation pathways [48,49]. Proper L-VGCC function is essential for neurotransmitter release, learning, and memory, whereas its dysregulation—as well as disturbances in GABAergic signaling—has been associated with schizophrenia [50]. Furthermore, vitamin D regulates nitric oxide synthases, thereby modulating nitric oxide (NO) levels, which are critical for synaptic vesicle release in GABAergic and glutamatergic neurons [51] (Figure 2). Taken together, these mechanisms suggest that vitamin D deficiency may impair synaptic plasticity and contribute to cognitive dysfunction [52].

## 4. The Neuroprotective Properties of Vitamin D

The potential beneficial effects of vitamin D on cognitive function across age groups and in patients with neurodegenerative diseases have been extensively investigated. Observational studies consistently report an association between low vitamin D levels and cognitive decline in older adults [53,54]. Individuals with vitamin D deficiency demonstrate lower scores on the Montreal Cognitive Assessment (MoCA), a screening tool for cognitive impairment [53,54]. In contrast, similar associations in young and middle-aged populations have generally not been established [55]. Nonetheless, some evidence suggests that higher vitamin D status may positively influence visual (non-verbal) memory in individuals with a history of early-life vitamin D deficiency or insufficient intake [56]. Substantial evidence supports the hypothesis that vitamin D_3_ plays a neuroprotective role by crossing the blood–brain barrier and interacting with vitamin D receptors, which contain both DNA- and ligand-binding domains [57]. Following ligand binding, VDR forms a heterodimer with the retinoid X receptor (RXR), translocates to the nucleus, and binds to vitamin D response elements (VDREs) in target gene promoters [58]. This genomic action gradually modulates brain function by upregulating the transcription of neurotrophic factors such as Brain-Derived Neurotrophic Factor (BDNF), Glial Cell Line-Derived Neurotrophic Factor (GDNF), and Ciliary Neurotrophic Factor (CNTF) in hippocampal neuronal stem cells [59]. Neurotrophic factors, including BDNF, facilitate synaptic plasticity, promote neuronal survival, and enhance cognitive performance [60]. Calcitriol exerts additional benefits on memory, motor function, and mood, which may be attributed to elevated expression of VDR and 1-α-hydroxylase in critical brain regions such as the hippocampus and substantia nigra [61]. Genomic neuroprotection in the aging brain is achieved not only through enhanced synthesis of neurotrophic factors but also through reduced expression of L-type voltage-gated calcium channels in hippocampal neurons [62]. This mechanism protects against excitotoxicity triggered by excessive *N*-methyl-D-aspartate (NMDA) and glutamate concentrations [63].

In addition to genomic actions, vitamin D_3_ exerts non-genomic effects by stabilizing intracellular calcium homeostasis via activation of kinase cascades, including PKC, PI3K, and MAPK pathways [64,65]. A membrane-associated receptor, PDIA3, mediates many of these rapid responses [66]. Upon activation, PDIA3 stimulates phospholipase A2 (PLA2) and phospholipase C (PLC), generating secondary messengers that activate PKC and ERK1/2 MAPKs [67]. These signaling pathways promote neuronal survival, differentiation, and synaptic plasticity [68]. PDIA3 functions complementarily to classical VDR, with PDIA3 primarily responsible for calcium mobilization and PKC activation, while VDR is essential for downstream CAMK2A activation [66]. Both receptors are required for a complete membrane-mediated response to 1,25(OH)_2_D_3_ [66].

Beyond vitamin D-specific pathways, neuroprotection arises from the interplay of biological and lifestyle factors. Physical activity, for instance, has consistently been associated with neuroprotective outcomes in both human and animal studies, in part through its effects on neurotrophin signaling, synaptic plasticity, and redox balance [69]. From a mitochondrial perspective, cortical spreading depression can induce adaptive upregulation of uncoupling protein-5 (UCP-5), enhancing resistance to metabolic stress [70]. While not directly related to vitamin D, such mechanisms provide context, showing how anti-inflammatory signaling, redox control, synaptic remodeling, and mitochondrial resilience converge to maintain neural health. Within this multidimensional framework, vitamin D status represents a modifiable factor that interacts with other exposures to shape neuroprotection.

## 5. Parkinson’s Disease

Parkinson’s disease (PD) is a progressive neurodegenerative disorder primarily caused by the loss of dopaminergic neurons in the substantia nigra pars compacta, leading to a significant reduction in striatal dopamine levels [71]. This dopaminergic deficit results in impaired neurotransmission within the nigrostriatal pathway, characterized by an imbalance between inhibitory GABAergic and excitatory cholinergic activity in the striatum [72]. Clinically, PD manifests with hallmark motor symptoms, including resting tremor, bradykinesia, muscular rigidity, and postural instability, as well as a wide spectrum of non-motor symptoms such as sleep disturbances, autonomic dysfunction, depression, and cognitive decline [71]. A pathological hallmark of PD is the accumulation of Lewy bodies, which are intracellular aggregates of misfolded α-synuclein protein within affected neurons [71]. These protein aggregates contribute to neuronal dysfunction and death through multiple mechanisms, including induction of oxidative stress, activation of neuroinflammatory pathways, impairment of protein degradation systems (ubiquitin–proteasome and autophagy–lysosome pathways), and mitochondrial dysfunction [71].

Vitamin D appears to exert a protective role in PD through several molecular and cellular mechanisms. Vitamin D receptors are abundantly expressed in the substantia nigra, particularly within dopaminergic neurons, suggesting a direct neurobiological role [73]. Upon binding to VDR, vitamin D modulates the transcription of numerous genes implicated in neuronal survival, differentiation, and synaptic plasticity [74]. Importantly, vitamin D stimulates the expression of neurotrophic factors such as nerve growth factor (NGF) and glial cell line-derived neurotrophic factor (GDNF), which promote dopaminergic neuron maintenance and repair [74]. In addition to its neurotrophic effects, vitamin D contributes to neuroprotection by reducing oxidative stress and excitotoxicity [74]. It suppresses the activity of inducible nitric oxide synthase (iNOS), thereby limiting excessive nitric oxide production and peroxynitrite formation, both of which are highly cytotoxic to neurons [74]. Vitamin D also regulates calcium homeostasis by modulating L-type voltage-gated calcium channels, preventing calcium overload that can trigger apoptosis in dopaminergic neurons [75]. Evidence also supports an anti-inflammatory role of vitamin D in PD. Another important pathway involves modulation of neuroinflammation. In an animal model, Calvello et al. demonstrated that vitamin D shifted microglial polarization in MPTP-induced Parkinson’s disease from a pro-inflammatory M1 phenotype toward an anti-inflammatory phenotype [76]. This was accompanied by reduced levels of iNOS, TNF-α, and IL-1β, and increased levels of IL-10, TGF-β, IL-4, and the markers CD163, CD204, and CD206 [76]. These results suggest that vitamin D may attenuate neuroinflammatory processes in degenerative conditions. Clinical studies have also demonstrated potential benefits. In one study, vitamin D supplementation was used as an adjunct to deep brain stimulation (DBS) therapy in Parkinson’s disease, leading to reduced IFN-γ levels [77]. Bytowska et al. further reported that 12 weeks of BMI-adjusted vitamin D_3_ supplementation in 29 patients with Parkinson’s disease improved motor function [77]. Significant improvements were observed in functional measures, including walking speed in the 6-Minute Walk Test and completion time in the Timed Up and Go Test. Serum 25(OH)D_3_ levels increased from 25.55 ± 8.94 ng/mL at baseline to 34.99 ± 12.27 ng/mL post-treatment (*p* < 0.0006). Although the sample size was small and objective measures of daily activity were lacking, these findings suggest that vitamin D_3_ supplementation may provide clinically relevant motor benefits [77].

Taken together, vitamin D supports dopaminergic neuron survival through neurotrophic, antioxidant, anti-inflammatory, and calcium-regulatory mechanisms. While promising, larger, biomarker-stratified clinical trials are needed to determine whether vitamin D supplementation can meaningfully alter the clinical course of PD or improve long-term patient outcomes.

## 6. Autism Spectrum Disorder

An analysis of the effects of vitamin D on cognitive function, synapse formation, anti-inflammatory activity, and neuroprotection has prompted investigation into its potential role in autism spectrum disorder (ASD). Individuals with ASD exhibit specific genotypes involving genes that regulate vitamin D metabolism and activity, including those encoding enzymes responsible for 25-hydroxylation, vitamin D receptors (VDR), and vitamin D-binding protein (DBP) [34,35]. Expression of several genes involved in DNA repair and apoptosis is closely associated with vitamin D status. Deficiency impairs DNA repair, limits apoptosis, and increases the risk of genetic mutations [78,79]. It has been hypothesized that early-life vitamin D deficiency may promote genetic mutations relevant to ASD pathogenesis, potentially outweighing pathogen-related factors [80]. Proteins influenced by vitamin D include Bcl-2-associated X protein (Bax), Growth Arrest and DNA-Damage-Inducible protein alpha (GADD45α), Tumor protein p53 (p53), RAD23 homolog B (RAD23B), proliferating cell nuclear antigen (PCNA), poly(ADP-ribose) polymerase (PARP), and death-associated protein 1α (DAP1α) [81].

**Figure 3:**
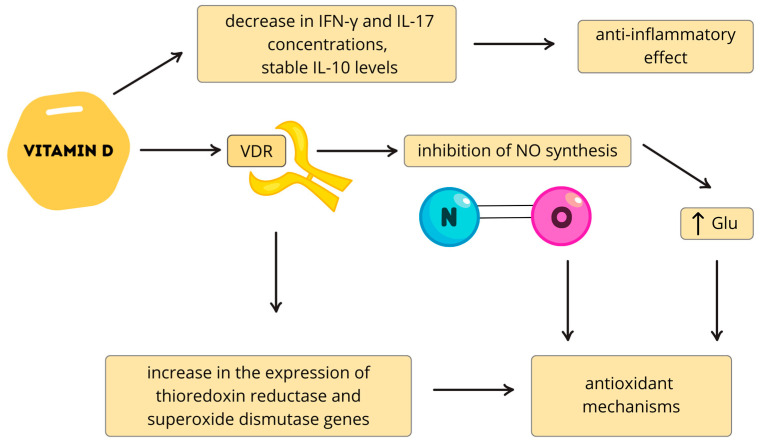
Vitamin D and antioxidant neuroprotection. By binding to vitamin D receptors (VDR), vitamin D inhibits nitric oxide (NO) synthesis, leading to increased (↑) glutathione levels and enhanced antioxidant processes that contribute to neuroprotection, while also upregulating thioredoxin reductase and superoxide dismutase to further support antioxidant capacity. Vitamin D also has an anti-inflammatory effect by reducing pro-inflammatory interleukins such as IL-6, while maintaining anti-inflammatory interleukins such as IL-10 at stable levels. This figure is original.

Vitamin D deficiency has been linked to disrupted neurotransmission affecting brain maturation, cortical organization, and behavior in ASD [82]. Supplementation has been shown to enhance GABA synthesis, support dopaminergic activity by increasing dopamine synthesis and metabolism, and upregulate GDNF, which promotes dopaminergic neuron survival [82]. Another protective mechanism involves inhibition of nitric oxide synthesis and enhancement of antioxidant defenses. Reduced NO production and increased glutathione are important neuroprotective mechanisms, often impaired in ASD [83,84]. Vitamin D also modulates the expression of genes encoding thioredoxin reductase and superoxide dismutase, further supporting antioxidant capacity [85,86]. Neuroinflammation is a well-documented feature of autism spectrum disorder, with elevated levels of pro-inflammatory cytokines such as IL-6, TNF-α, and IFN-γ frequently reported in patients [87]. These cytokines are known to impair cognitive function. Vitamin D supplementation has been shown to inhibit cytokine production, thereby exerting potential anti-inflammatory and neuroprotective effects [88,89]. The study by Walawska-Hrycek et al. showed that with increased vitamin D_3_ supplementation, there was a decrease in the concentration of pro-inflammatory cytokines such as IFN-γ and IL-17, while interleukin IL-10 (an anti-inflammatory cytokine) levels remained stable [89] (Figure 3).

In a randomized, double-blind, placebo-controlled trial involving 43 children with ASD (mean age 8.91 ± 2.87 years; 7 girls, 36 boys), supplementation with vitamin D_3_ at 300 IU/kg/day (up to 6000 IU/day) for 15 weeks led to a significant increase in serum 25(OH)D levels compared with baseline (*p* = 0.001) and placebo [90]. Improvements were observed in core ASD symptoms as assessed by the Childhood Autism Rating Scale (CARS) and Autism Treatment Evaluation Checklist (ATEC). Although no significant changes were detected in serotonin or IL-6 levels, clinical outcomes improved significantly in the vitamin D group (*p* = 0.021 for CARS; *p* = 0.020 for ATEC) [90] At baseline, more than 86% of participants were vitamin D deficient. While exact point changes in CARS and ATEC were not consistently reported, the significant *p*-values indicate measurable clinical benefits [90]. Subgroup analyses from other studies suggest that inflammatory status may influence treatment response. For example, children with elevated IL-1β levels demonstrated greater improvements when treated with vitamin D, omega-3 long-chain polyunsaturated fatty acids (LCPUFAs), or a combination of both [90,91]. The most pronounced improvements—in areas such as social communication and awareness—were observed in children with elevated IL-1β, regardless of intervention type [90,91]. These findings imply that omega-3 LCPUFAs may act synergistically with vitamin D, particularly in children with active inflammation. However, the absence of clearly defined IL-1β thresholds limits the precision of these observations and introduces variability across studies.

Maternal vitamin D status has also been implicated in ASD risk. Multiple meta-analyses demonstrate a significant association between higher maternal 25(OH)D levels and a reduced incidence of autism-related traits in offspring [92,93,94,95]. Comparisons between the highest and lowest prenatal vitamin D categories revealed improved cognitive outcomes in children, supporting the importance of adequate maternal supplementation during pregnancy. A prospective study further investigated the effects of high-dose vitamin D supplementation during pregnancy on the risk of ASD and attention-deficit/hyperactivity disorder (ADHD) [96]. Mothers received either the standard 400 IU/day or an elevated 2800 IU/day dose from 24 weeks of gestation until one week postpartum [96]. The higher maternal dose was associated with a reduced risk of ASD and ADHD and with milder autism symptoms in affected children [96]. Importantly, high maternal vitamin D intake was not linked to an increased risk of adverse neuropsychiatric outcomes [96]. The study noted several limitations, including unmeasured confounders such as parental mental health, and emphasized the need to define the optimal timing and dosage of prenatal supplementation. Although these findings are encouraging, the overall evidence remains inconclusive. Many clinical studies are limited by modest sample sizes, heterogeneity in outcomes, and the lack of biomarker stratification (e.g., baseline 25(OH)D or cytokine levels). To determine which ASD subgroups benefit most, adequately powered, biomarker-guided clinical trials are urgently needed.

## 7. Alzheimer’s Disease

Alzheimer’s disease (AD) is the most common cause of dementia worldwide and represents a major public health challenge due to its progressive course and lack of curative treatment [97]. Clinically, AD is characterized by gradual cognitive decline, memory impairment, and deficits in executive and behavioral functions [97]. Pathologically, the disease is defined by two hallmark features: extracellular amyloid plaques and intracellular neurofibrillary tangles, primarily affecting cortical and limbic regions of the brain [98]. The formation of amyloid plaques results from abnormal proteolytic processing of the amyloid precursor protein (APP) by β-secretase and γ-secretase, which generates amyloid-β (Aβ) peptides, predominantly Aβ40 and the more aggregation-prone Aβ42 [97]. These monomers oligomerize and accumulate, ultimately forming senile plaques. Concurrently, tau proteins undergo hyperphosphorylation, leading to the development of neurofibrillary tangles that disrupt microtubule stability and impair axonal transport [99]. Beyond these structural changes, high concentrations of Aβ in the central nervous system stimulate microglial activation, initiating a sustained neuroinflammatory response [100]. This immune dysregulation, combined with oxidative stress and mitochondrial dysfunction, contributes to synaptic loss and progressive neuronal death, which underlies the clinical manifestations of AD [101].

The association between vitamin D and Alzheimer’s disease has also been explored [102]. One study evaluated the effects of 12 months of oral vitamin D therapy on cognitive function and amyloid beta biomarkers, as amyloid deposition plays a central role in Alzheimer’s pathogenesis [103]. Cognitive performance was assessed at three time points using the Wechsler Adult Intelligence Scale-Revised (WAIS-RC), the Activity of Daily Living (ADL) Scale, and the Mini-Mental State Examination (MMSE), while blood samples were analyzed for mRNA expression of amyloid precursor protein (APP), β-secretase 1 (BACE1), presenilin-1 (PS1), presenilin-2 (PS2), and amyloid beta and vitamin D_3_ concentrations [103]. The intervention resulted in significant reductions in amyloid beta markers, including Aβ42, APP, BACE1, APP mRNA, and BACE1 mRNA, as well as improvements in multiple cognitive domains, such as information, digit span, vocabulary, block design, picture arrangement, and arithmetic [103]. Meta-analyses support these findings, showing a correlation between vitamin D_3_ deficiency (serum 25(OH)D < 10 ng/mL and < 20 ng/mL) and an increased risk of Alzheimer’s disease and dementia [104,105]. These results suggest that maintaining adequate vitamin D_3_ levels may protect cognitive function in older adults. Optimal concentrations for prevention vary, with estimates of >40.1 nmol/L for Alzheimer’s disease and 77.5–100 nmol/L for dementia [104].

In addition, patients with Alzheimer’s disease show increased levels of vitamin D-binding protein (DBP) in cerebrospinal fluid (CSF) [106]. DBP has multiple binding sites and is known to scavenge toxic actin, raising the hypothesis that it may influence amyloid beta levels [107]. Experimental work demonstrated that DBP directly binds Aβ and suppresses Aβ-related pathology in vitro and in vivo. Notably, intracerebroventricular injection of DBP improved Aβ-induced cognitive dysfunction and protected against hippocampal synapse loss in mice [108]. Research findings indicate that Aβ may inhibit VDR expression in cortical neurons, which may contribute to neurodegenerative processes [109]. When the number of VDRs is reduced, there is an increase in the expression of the voltage-sensitive calcium channel A1C type L (LVSCC-A1C), while the level of nerve growth factor (NGF) decreases [62]. This disruption suggests that VDR suppression makes neurons more susceptible to aging and neurodegeneration, particularly in the context of Aβ toxicity [110].

Another area of investigation is the effect of vitamin D_3_ on telomere length and oxidative stress in Alzheimer’s disease and mild cognitive impairment (MCI) [111]. A study evaluating one year of vitamin D_3_ supplementation showed beneficial effects on leukocyte DNA telomere length and oxidative stress markers [111]. Previous research has linked longer telomeres to better cognitive outcomes in MCI and Alzheimer’s disease [112]. Telomeres are protective chromosomal end regions, preventing damage during replication [113]. Vitamin D_3_ appears to help maintain telomere integrity, preventing premature shortening and stabilizing their structure [114]. Telomere attrition can activate DNA damage responses (DDR), reflected by increased oxidative stress markers [115]. In this study, serum levels of 8-oxo-2′-deoxyguanosine (8-OXO-dG), 8-oxoguanine DNA glycosylase (OGG1) mRNA, and P16INK4a mRNA were measured, as these markers indicate oxidative DNA damage and stress [116,117]. Following vitamin D_3_ therapy, oxidative stress markers decreased, leukocyte telomeres lengthened, and cognitive scores improved [116,117]. These findings reinforce vitamin D_3_’s neuroprotective potential in age-related cognitive decline. The evidence remains limited by small sample sizes, short intervention periods, and variability in vitamin D_3_ dosing and baseline 25(OH)D levels. Leukocyte telomere length serves only as a peripheral marker and may not fully capture neuronal processes. Longer-term, larger trials are needed to confirm these effects.

## 8. Multiple Sclerosis

Several studies have examined the effect of vitamin D supplementation on the progression of multiple sclerosis (MS), particularly the relapsing–remitting form (RRMS) [118]. A history of infectious mononucleosis is considered a potential risk factor for MS, and vitamin D supplementation has been shown to reduce antibody levels against Epstein–Barr virus (EBV) [119,120]. This reduction may lower the risk of a single clinically isolated syndrome (CIS) progressing to RRMS, as higher antibody titers are associated with greater progression risk [121]. A beneficial effect of vitamin D treatment in RRMS has been demonstrated in other studies, where increased 1,25(OH)_2_D levels correlated with reduced relapse frequency [122]. Short-term vitamin D supplementation (two months) also enhanced the activity of DNA repair genes in MS patients [123]. Vitamin D status has been proposed as a potential marker of higher cognitive reserve; in one study, patients with higher 1,25(OH)_2_D levels one year after CIS achieved Paced Auditory Serial Addition Test (PASAT) scores 11 years later that were above the cohort mean [124]. Early vitamin D administration may also help maintain immune balance during disease progression. In RRMS patients receiving interferon beta (IFN-β), no reduction in anti-inflammatory Th L4^+^ cells was observed compared to controls, suggesting that vitamin D supplementation stabilized this population [125]. In another study, RRMS patients with vitamin D deficiency were supplemented for two months to assess effects on oxidative stress-related genes (*NRF2*) and DNA repair genes (*OGG1*, *MYH*, *MTH1*) [123]. *NRF2* regulates genes containing antioxidant response elements (ARE), while *OGG1*, *MYH*, and *MTH1* encode enzymes that eliminate oxidative nucleotides [126,127]. Clinically significant upregulation of these genes occurred only in patients whose serum vitamin D levels increased with supplementation.

MS is characterized by elevated pro-inflammatory cytokines, including IL-2, IL-6, and TNF-α, making their modulation by vitamin D_3_ of particular interest [128]. Although vitamin D is generally associated with a Th1→Th2 shift and reduced IL-2, IL-6, and IFN-γ expression, evidence from RRMS cohorts shows that its immunomodulatory effects can be context-dependent, with some studies reporting increased IL-6 gene expression potentially reflecting an indirect suppression of pathogenic IL-17-driven pathways. One study found increased expression of IL-6-encoding genes, possibly due to the inverse relationship between IL-6 and IL-17 production by Th17 lymphocytes, as IL-17 plays a key role in MS pathology [129,130]. Importantly, vitamin D_3_ directly inhibits Th17 cells and IL-17 transcription [89]. Potential contributors to elevated IL-6 levels include interactions with sex hormones, seasonal variation in sampling, and concomitant medications. Despite these findings, vitamin D_3_ appears to exert stronger effects on pro-inflammatory pathways overall, decreasing cytokine production by Th1, Th9, and Th22 lymphocytes [131,132].

## 9. Conclusions

The reviewed evidence indicates that vitamin D_3_ exerts broad neuroprotective effects across aging, neurodegenerative diseases, and neurodevelopmental disorders. Its actions are mediated through both genomic and rapid non-genomic pathways that collectively modulate calcium homeostasis, neurotrophin signaling, antioxidant defenses, inflammatory cascades, neurotransmission, and DNA repair mechanisms.

In Alzheimer’s disease, supplementation has been shown to reduce amyloid-β burden, improve cognitive performance, lengthen leukocyte telomeres, and decrease oxidative stress. Preclinical studies additionally suggest that delivery of vitamin D-binding protein (DBP) may enhance cognition and synaptic preservation, supporting the rationale for exploring DBP-based therapies.

In Parkinson’s disease, vitamin D_3_ contributes to the survival of dopaminergic neurons by regulating calcium channels, suppressing oxidative and inflammatory stress, and enhancing neurotrophic support. Clinical data further demonstrate that supplementation may improve motor outcomes and functional capacity, particularly when combined with existing therapies such as deep brain stimulation.

In autism spectrum disorder (ASD), sufficient vitamin D_3_ levels have been linked to improved behavioral and cognitive outcomes. Supplementation modulates genes involved in antioxidant defense, protein synthesis, apoptosis, and neurotransmission (e.g., GABAergic and dopaminergic signaling, GDNF expression). While vitamin D alone may not fully prevent ASD progression, clinical trials suggest benefits—especially in children with elevated baseline inflammation or when combined with omega-3 long-chain polyunsaturated fatty acids. Moreover, maternal supplementation during pregnancy has been associated with a reduced risk of ASD and ADHD, underscoring the importance of optimal vitamin D status from the fetal period onward.

In multiple sclerosis (MS), vitamin D_3_ influences immune regulation by decreasing pro-inflammatory cytokines (e.g., IL-17, Th1, Th9, Th22 responses) while enhancing DNA repair and antioxidant capacity via pathways such as OGG1, MYH, MTH1, and NRF2. Baseline vitamin D status may also predict cognitive resilience in patients with MS.

Taken together, these findings highlight vitamin D_3_ as a promising adjunct in the prevention and management of diverse central nervous system disorders (Table 2).

Future research should focus on the application of precision medicine, integrating the assessment of vitamin D status with the analysis of genetic polymorphisms (CYP2R1, CYP27B1, VDR), which would enable personalized dosing and increase the effectiveness of supplementation. Another important area of development is combination therapy strategies, in which vitamin D_3_ would be combined with deep brain stimulation (DBS), neurotrophic factors (NGF, GDNF), or biological anti-inflammatory drugs to achieve a synergistic effect. Another innovative area is the development of new delivery systems, such as vitamin D-binding protein (DBP)-based drugs or nanocarriers targeting the central nervous system, which could increase bioavailability in the brain and potentially improve neuroprotective effects. It is also worth considering interventions in early life, including prenatal supplementation and pediatric studies to assess the long-term impact on brain development and the prevention of neurodevelopmental disorders. Additionally, the use of digital tools—such as wearable devices and artificial intelligence algorithms—may enable real-time monitoring of vitamin D levels, sunlight exposure, and patients’ motor and cognitive functions, supporting early detection of deterioration and rapid therapeutic intervention.

An innovative aspect of this review is its comprehensive synthesis of emerging molecular mechanisms and translational insights linking vitamin D_3_ to neuroprotection across a broad spectrum of neurodegenerative and neurodevelopmental disorders. The review integrates both genomic and non-genomic pathways of vitamin D_3_ action, highlighting novel targets such as PDIA3-mediated rapid signaling and the modulation of L-type voltage-gated calcium channels, which are essential for synaptic plasticity. It also addresses the potential of combining vitamin D_3_ therapy with advanced clinical interventions, including deep brain stimulation, and explores the use of vitamin D-binding protein nanocarriers to enhance central nervous system (CNS) bioavailability. Importantly, it emphasizes precision medicine approaches by considering genetic polymorphisms that affect vitamin D metabolism, thereby enabling tailored supplementation strategies. By incorporating contemporary clinical trial data on Parkinson’s disease, Alzheimer’s disease, autism spectrum disorder, and multiple sclerosis, and by presenting a forward-looking perspective on the integration of digital health tools for real-time monitoring, this work establishes itself as novel, integrative, and impactful for the future of neurotherapeutics.

However, considerable heterogeneity across studies—in dosing regimens, achieved 25(OH)D levels, population characteristics, and outcome measures—limits direct comparison. Future research should prioritize longitudinal population-based cohorts and well-powered randomized controlled trials. Particular attention should be given to genetic polymorphisms regulating vitamin D metabolism, as these may explain interindividual differences in treatment responsiveness and inform more personalized supplementation strategies. Despite these limitations, the collective body of evidence suggests that maintaining sufficient vitamin D_3_ status represents a modifiable factor with significant potential to support brain health, delay neurodegeneration, and improve functional outcomes across the lifespan.

## Figures and Tables

**Table 1:** Table summarizing the effects of vitamin D_3_ on specific tissues in the body. CaATPase—Calcium-transporting ATPase, PMCA1b—Plasma Membrane Calcium ATPase isoform 1b, VDR—Vitamin D Receptor, PTH—Parathyroid Hormone, Calbindin-D28k—Calcium-binding protein D28k, Th1—T helper type 1, Th2—T helper type 2, Th17—T helper type 17, Treg—Regulatory T cells, TRH—Thyrotropin-Releasing Hormone, TSH—Thyroid-Stimulating Hormone, Cai—Intracellular calcium, IP3—Inositol 1,4,5-triphosphate.

Target Tissue	Response
Intestine	stimulation of transcellular calcium transport in duodenum and caecum; inducing basolateral CaATPase, PMCA1b [1]
Bone	optimal endochondral bone formation, increasing the number and activity of osteoclasts [2,3]
Kidney	regulation of calcium (distal tubule) and phosphate (proximal tubule) transport, stimulating VDR, calcium pump and calbindin [4,5,6,7]
Parathyroid gland	inhibition of production of parathormon—suppression of the PTH promoter [8]
Pancreatic-cells	stimulation of insulin secretion; calbindin-D28k modulates depolarization-stimulated insulin release [9,10]
Skin	calcium induced regulation of keratinocyte differentiation; stimulation of wound healing, regulation of hair follicle cycle [11,12,13]
Immune system	stimulation of cathelicidin, inhibition of the maturation of dendritic cells; reduction in T cell proliferation and shifting the balance of T cell differentiation from the Th1 and Th17 pathways to Th2 and Treg pathways [14,15,16]
Heart	increase in contractility of cardiac muscle, stimulation of calcium uptake; inhibition of atrial natriuretic factor’s promoter [17,18]
Pituitary gland	increase in the TRH-stimulated TSH secretion through increased Cai and IP3 production [19]
Liver	stimulation of hepatic regeneration [20]
Lung	stimulation of type TT epithelial pneumocytes through increased phospholipid production and surfactant release [21]

**Table 2:** Summary of Vitamin D_3_ Actions and Supplementation Outcomes in Selected Neuropsychiatric and Neurodegenerative Disorders.

Disease	Mechanism of Action of Vitamin D_3_	Effect of Supplementation
Parkinson’s Disease	modulation of microglia towards an anti-inflammatory phenotype [76]; decrease in pro-inflammatory markers and increase in anti-inflammatory markers [76]	Improve motor outcomes and functional capacity [77]
Autism Spectrum Disorder	Increased GABA synthesis, support for dopaminergic activity, and increased GDNF levels [82] Increased glutathione production and decreased NO synthesis [83,84] Inhibition of pro-inflammatory cytokine production [88]	Improvement in social functioning, communication, better results in CARS and ATEC tests [90]
Alzheimer’s Disease	Reduced beta-amyloid marker concentration [103] Leukocyte telomere elongation [111] Telomere structure stabilization [114] Reduced oxidative stress [111,116]	Improved cognitive function [103]
Multiple Sclerosis	Reduction in EBV antibody levels [119,120]; Inhibition of Th17 lymphocyte activity and IL-17 transcription [89]; reduction in cytokine synthesis produced by: Th1, Th9, and Th22 [131,132]	Reduced risk of CIS → RRMS progression [119,120,121] Reduced relapse frequency in RRMS [118]

## Abbreviations

25(OH)D/25(OH)D_3_	25-Hydroxyvitamin D
1,25(OH)_2_D/1,25(OH)_2_D_3_	1,25-Dihydroxyvitamin D_3_ (Calcitriol)
AD	Alzheimer’s Disease
ADHD	Attention-Deficit/Hyperactivity Disorder
APC	Antigen-Presenting Cell
APP	Amyloid Precursor Protein
Aβ/Aβ40/Aβ42	Amyloid-β Peptides
ARE	Antioxidant Response Element
ASD	Autism Spectrum Disorder
ATEC	Autism Treatment Evaluation Checklist
Bax	Bcl-2-Associated X Protein
BDNF	Brain-Derived Neurotrophic Factor
BMI	Body Mass Index
Cai	Intracellular Calcium
CAMK2A	Calcium/Calmodulin-Dependent Protein Kinase II Alpha
CARS	Childhood Autism Rating Scale
CaATPase	Calcium-Transporting ATPase
CD4+	Cluster of Differentiation 4 Positive T Cells
CIS	Clinically Isolated Syndrome
CNS	Central Nervous System
CNTF	Ciliary Neurotrophic Factor
CSF	Cerebrospinal Fluid
CYP2R1	Cytochrome P450 Family 2 Subfamily R Member 1
CYP24A1	Cytochrome P450 Family 24 Subfamily A Member 1
CYP27B1	Cytochrome P450 Family 27 Subfamily B Member 1
DAP1α	Death-Associated Protein 1 Alpha
DBP	Vitamin D-Binding Protein
DBS	Deep Brain Stimulation
DDR	DNA Damage Response
EBV	Epstein–Barr Virus
ERK1/2 MAPKs	Extracellular Signal-Regulated Kinases 1/2, Mitogen-Activated Protein Kinases
FGF23	Fibroblast Growth Factor 23
GABA	Gamma-Aminobutyric Acid
GADD45α	Growth Arrest and DNA-Damage-Inducible Protein Alpha
GDNF	Glial Cell Line-Derived Neurotrophic Factor
IFN-β	Interferon Beta
IFN-γ	Interferon Gamma
IL/IL-1β/IL-2/IL-4/IL-6/IL-10/IL-17	Interleukins
iNOS	Inducible Nitric Oxide Synthase
IP_3_	Inositol 1,4,5-Triphosphate
IU	International Units
LCPUFA	Long-Chain Polyunsaturated Fatty Acids
L-VGCCs	L-Type Voltage-Gated Calcium Channels
LVSCC-A1C	L-Type Voltage-Sensitive Calcium Channel A1C
MAPK	Mitogen-Activated Protein Kinase
MCI	Mild Cognitive Impairment
MoCA	Montreal Cognitive Assessment
MS	Multiple Sclerosis
MPTP	1-Methyl-4-Phenyl-1,2,3,6-Tetrahydropyridine
MYH	MutY DNA Glycosylase
MTH1	MutT Homolog 1
NGF	Nerve Growth Factor
NMDA	N-Methyl-D-Aspartate
NO	Nitric Oxide
NRF2	Nuclear Factor Erythroid 2-Related Factor 2
OGG1	8-oxoguanine DNA Glycosylase
P16INK4a	Cyclin-Dependent Kinase Inhibitor 2A
PARP	Poly(ADP-Ribose) Polymerase
PASAT	Paced Auditory Serial Addition Test
PCNA	Proliferating Cell Nuclear Antigen
PD	Parkinson’s Disease
PDIA3	Protein Disulfide-Isomerase A3
PI3K	Phosphatidylinositol 3-Kinase
PKC	Protein Kinase C
PLA2	Phospholipase A2
PLC	Phospholipase C
PMCA1b	Plasma Membrane Calcium ATPase Isoform 1b
PS1/PS2	Presenilin-1/Presenilin-2
PTH	Parathyroid Hormone
RAD23B	RAD23 Homolog B
RRMS	Relapsing–Remitting Multiple Sclerosis
RXR	Retinoid X Receptor
Th1/Th2/Th17/Th9/Th22	T Helper Cell Subtypes
TGF-β	Transforming Growth Factor Beta
TNF-α	Tumor Necrosis Factor Alpha
TRH	Thyrotropin-Releasing Hormone
TSH	Thyroid-Stimulating Hormone
Treg/Tregs	Regulatory T Cell(s)
UCP-5	Uncoupling Protein-5
VDR	Vitamin D Receptor
VDRE	Vitamin D Response Element
8-OXO-dG	8-oxo-2′-deoxyguanosine
